# Cerebral Venous Sinus Thrombosis Mimicking Tumor Hemorrhage: Successful Anticoagulation in a Resource-Limited Setting

**DOI:** 10.7759/cureus.83918

**Published:** 2025-05-11

**Authors:** H.K.D. Kasun Prabasara, Kithsiri Niwunhella, Anomali Vidanagamage, Hirushaka De Silva, Muditha Dahanayaka

**Affiliations:** 1 Medicine, District General Hospital - Hambantota, Hambantota, LKA; 2 Neurology, District General Hospital - Hambantota, Hambantota, LKA; 3 Anaesthesiology, District General Hospital - Hambantota, Hambantota, LKA; 4 Radiology, District General Hospital - Hambantota, Hambantota, LKA

**Keywords:** anticoagulation, case report. venous stroke., cerebral venous sinus thrombosis, cerebral venous thrombosis cvt, cvst, expressive aphasia, hemorrhagic stroke, tumor bleed, tumor mimic, venous infarction

## Abstract

Cerebral venous sinus thrombosis (CVST) is a rare yet potentially reversible etiology of stroke that frequently presents with nonspecific symptoms, contributing to delayed diagnosis. Aphasia, commonly associated with arterial infarcts, may also occur in CVST when thrombosis involves cortical veins responsible for language function. We report a case of a middle-aged female who presented with expressive aphasia and headache. Initial non-contrast computed tomography (CT) revealed hemorrhagic changes suggestive of tumor-related bleed. However, subsequent magnetic resonance venography (MRV) confirmed CVST involving the left transverse sinus. Anticoagulation with low molecular weight heparin (LMWH) was initiated despite the presence of hemorrhagic venous infarction and was well tolerated, leading to progressive clinical improvement. This case underscores the diagnostic challenges posed by CVST when imaging mimics neoplastic hemorrhage and highlights the importance of early venographic imaging. Timely recognition and initiation of anticoagulation, even in the presence of hemorrhage, remain critical to achieving favorable outcomes.

## Introduction

Cerebral venous thrombosis (CVT) is a relatively rare form of stroke caused by thrombosis in the cerebral veins and dural sinuses, accounting for approximately 0.5-1% of all strokes. It typically affects young adults, particularly females. According to the largest cohort study to date - the International Study on Cerebral Venous and Dural Sinus Thrombosis (ISCVT) - 78% of the 624 patients studied were under the age of 50 [1,2]. CVT is a multifactorial disorder with diverse underlying risk factors. Data from ISCVT and other large cohorts indicate that the most common and strongly associated risk factors include oral contraceptive use, pregnancy and the puerperium, and inherited or acquired prothrombotic states. Oral contraceptives alone account for over half the cases among women, while prothrombotic conditions are identified in over a third of all patients [1]. Additional contributors include malignancies, parameningeal infections, certain medications (e.g., androgens, IV immunoglobulin, and chemotherapeutic agents), mechanical factors (e.g., lumbar puncture, spontaneous intracranial hypotension), and systemic diseases such as systemic lupus erythematosus (SLE), inflammatory bowel disease, and thyroid disorders [1]. Notably, in about 12.5% of patients, no identifiable risk factor is found, underscoring the need for a high index of suspicion in atypical presentations [1].

CVT can involve various components of the cerebral venous system. The superior sagittal sinus is the most frequently affected, seen in about 62% of cases. This is followed by the transverse sinus (41-45%), the straight sinus (18%), cortical veins (17%), the deep venous system (11%), and the internal jugular vein (12%). The sigmoid sinus, although commonly affected in conjunction with the transverse sinus, was not separately quantified in terms of prevalence [1]. Unlike arterial strokes, CVT presents with a wide array of nonspecific symptoms, which often delays diagnosis [1,3]. Clinical symptoms generally fall into two categories: those due to increased intracranial pressure from impaired venous drainage, and those resulting from focal brain injury caused by venous infarction, ischemia, or hemorrhage [2]. The most common presenting symptom is headache, occurring in nearly 90% of patients in the ISCVT study. It is often diffuse and progressive, sometimes mimicking thunderclap or migraine headaches. Up to 25% of patients may present with isolated headache, with no focal neurological deficits or papilledema, complicating the diagnostic process [2].

CVT should be considered in patients with headache and papilledema or diplopia due to sixth nerve palsy, even when other neurological signs are absent, as these features can mimic idiopathic intracranial hypertension. When focal brain injury occurs, symptoms such as hemiparesis, aphasia, sensory deficits, and other cortical signs are common. Rarely, psychosis can occur alongside these signs [2,4]. The clinical manifestations vary depending on the location of the thrombus. Superior sagittal sinus thrombosis - the most frequent type - typically presents with headache, papilledema, motor deficits, and seizures. Lateral (transverse) sinus thrombosis is often linked to middle ear infections and may present with fever, ear discharge, mastoid tenderness, raised intracranial pressure, and occasionally, hemianopia, aphasia, or contralateral weakness. Deep cerebral venous thrombosis, which affects approximately 16% of CVT patients, often causes thalamic or basal ganglia infarcts, leading to rapid neurological decline and altered consciousness. Cortical vein thrombosis is a rare condition that may manifest with focal cortical symptoms or temporal lobe hemorrhage, particularly when the vein of Labbé - a major anastomotic vein draining the lateral temporal lobe into the transverse sinus - is involved [2].

Non-contrast CT (NCCT) is often the initial imaging modality in suspected CVT, but it has limited sensitivity. Up to 25-30% of patients may have normal NCCT findings, particularly in the early stages. One direct sign is the “cord sign,” representing a hyperdense thrombus in a dural sinus or cortical vein, seen in about 25% of cases. Its sensitivity varies between 25% and 64%, but it has high specificity (97%), making it a reliable but not frequent finding [5]. The “dense vein sign” describes a hyperattenuating thrombus within a cortical or dural sinus on NCCT, especially in the superior sagittal sinus. When contrast is used, it may produce a “delta sign” or “triangle sign” due to central filling defects surrounded by enhancing dura. The “cashew nut sign” is another distinctive radiological feature- a concave, small juxtacortical hemorrhage, highly specific (98%) for CVT, though with low sensitivity (26%) [5,6].

Venous infarction occurs in up to 50% of CVT patients and results from obstructed venous outflow, increasing venous pressure, and reducing perfusion. Unlike arterial infarcts, these are often hemorrhagic and may be reversible with prompt anticoagulation, reflecting a unique clinical and therapeutic profile [2,7]. In some studies, NCCT sensitivity for CVT reaches 100% in expert hands, though this likely includes indirect signs and introduces bias. Specificity in these cases is around 83% [8]. Isolated cortical vein thrombosis is difficult to detect with NCCT, having only 25% sensitivity but 100% specificity [9]. NCCT may miss isodense thrombi, making confirmatory imaging with CT venography (CTV) or magnetic resonance venography (MRV) essential.

CTV is a contrast-enhanced technique offering detailed imaging of the intracranial venous system. It has a reported sensitivity of 75-100% and specificity of 81-100%, depending on thrombus location [10,11]. It is effective in detecting filling defects, such as the “empty delta sign” in the superior sagittal sinus. While CTV performs well overall, its sensitivity is reduced in deeper venous locations like the vein of Galen and right transverse sinus [12]. Nevertheless, studies show that CTV is comparable to MRV in diagnostic accuracy, making it an excellent first-line tool, particularly in emergency settings [10,11]. However, it may not be as sensitive in isolated cortical vein or deep system thrombosis [11].

MRV is considered the gold standard for CVT diagnosis due to its superior imaging quality and non-invasive nature. It can detect thrombi in both dural sinuses and cortical veins [5,9,11]. Techniques include time-of-flight (TOF), phase-contrast, and contrast-enhanced MRV, with the latter offering better image quality, especially in areas of slow or turbulent flow [2]. MRV can also evaluate parenchymal complications like infarction or hemorrhage, and is ideal for young or pregnant patients due to the lack of ionizing radiation. However, limitations include reduced availability in emergencies, longer acquisition time, and susceptibility to motion artifacts. Nonetheless, MRV remains the diagnostic modality of choice when CVT is strongly suspected.

Laboratory work-up should include thrombophilia screening, complete blood count, inflammatory markers, and etiology-specific tests such as those for infection, pregnancy, or COVID-19 [13]. Anticoagulation is the cornerstone of CVT treatment, even when hemorrhagic infarction is present. While large randomized trials are limited, meta-analyses and observational studies confirm that anticoagulation reduces mortality and long-term disability. LMWH may be more effective and equally safe compared to UFH [14,15].

Endovascular therapy, including thrombectomy or local thrombolysis, is considered in patients who deteriorate despite anticoagulation. Although high recanalization rates have been reported, the Thrombolysis or Anticoagulation for Cerebral Venous Thrombosis (TO-ACT) trial did not demonstrate a mortality benefit [16,17]. Management of acute complications like elevated intracranial pressure, cerebral edema, and seizures includes ICU care, osmotic agents, and decompressive hemicraniectomy in select cases. Steroids are not recommended unless there is an associated inflammatory condition. Seizure prophylaxis is advised for patients with seizures and supratentorial lesions on imaging [18].

Post-acute anticoagulation usually continues for three to 12 months, depending on risk factor profiles. DOACs are often preferred due to convenience and fewer interactions, but warfarin is used in cases of antiphospholipid syndrome, active cancer, or renal impairment, with a target INR of 2-3. Despite an early mortality rate of 5%, long-term outcomes are generally good, with approximately 80% of patients achieving full recovery. The CVT Risk Score can help identify patients at greater risk of poor outcomes and guide long-term management strategies.

## Case presentation

A 48-year-old female presented with expressive aphasia of one day’s duration, preceded by a nonspecific, dull headache for two days. On the day of admission, she also experienced two episodes of vomiting, but denied fever, seizures, photophobia, or neck stiffness. The aphasia had a sudden onset, gradually worsening over eight to 12 hours, and was not associated with limb weakness, facial asymmetry, vertigo, dizziness, or visual or auditory disturbances. She had no history of similar neurological symptoms in the past, nor any known history of migraine, seizure disorders, or connective tissue disease. There was no prior history of thrombotic events, pregnancy losses, oral contraceptive pill use, or symptoms suggestive of SLE (e.g., photosensitive rash, oral ulcers, joint pain, or Raynaud phenomenon). She denied recent weight loss, loss of appetite, or evening fevers. Her vaccination history was up to date, and she reported no known drug or food allergies. Her past medical, surgical, and family history was unremarkable.

On general examination, the patient appeared ill but was not pale or icteric, and there were no peripheral stigmata suggestive of connective tissue disorders. On neurological examination, she was alert and well oriented. Gross papilledema was noted on fundoscopy. All cranial nerves were intact. In the upper limbs, tone was normal, but there was mild weakness in the right upper limb, with a power of 4/5. Reflexes and sensation were normal in both upper limbs. Examination of the lower limbs revealed that tone, power, reflexes, and sensation were all within normal limits. However, plantar responses were upgoing bilaterally. Her pulse rate was 64 beats per minute, and her blood pressure was 110/70 mmHg. No murmurs were heard on cardiovascular examination. Both respiratory and abdominal examinations were normal.

The complete blood count (Table 1) demonstrated generally preserved red cell parameters. Hemoglobin was within the normal range at 11.3 g/dL (reference: 11.0-16.0 g/dL), with a hematocrit of 37.4% (reference: 37.0-54.0%) and normal mean corpuscular volume (MCV) of 85.1 fL (reference: 80.0-100.0 fL). Red cell indices, including mean corpuscular hemoglobin (MCH) at 28.5 pg (reference: 27.0-34.0 pg), were also within normal limits. Although the RBC count was slightly below the reference range at 3.25 × 10¹²/L (reference: 3.5-5.5 × 10¹²/L), this isolated finding did not indicate overt anemia. The platelet count was elevated at 478 × 10³/µL (reference: 150-450 × 10³/µL), which may suggest a reactive thrombocytosis in the context of a systemic thrombotic or inflammatory process. The total white blood cell count remained within normal limits at 8.72 × 10⁹/L (reference: 4.00-10.00 × 10⁹/L), but differential counts revealed a marked neutrophilia (89.1%) and relative lymphopenia (9.4%). This pattern is frequently observed in acute-phase responses and may reflect systemic stress, inflammation, or infection secondary to cerebral venous sinus thrombosis (CVST).

**Table 1:** Hematology MCH - mean corpuscular hemoglobin; MCHC - mean corpuscular hemoglobin concentration; MCV - mean corpuscular volume; RBC count - red blood cell count; WBC - white blood cell count

Test	Result	Reference Range	Interpretation
Hemoglobin (Hb)	11.3 g/dL	11.0-16.0 g/dL	Normal
Hematocrit (HCT)	37.4%	37.0-54.0%	Normal
RBC count	3.25 × 10¹²/L	3.5-5.5 × 10¹²/L	Normal
MCV	85.1 fL	80.0-100.0 fL	Normal
MCH	29.5 pg	27.0-34.0 pg	Normal
MCHC	34.9 g/dL	32.0-36.0 g/dL	Normal
Platelet count	478 × 10³/µL	150-450 × 10³/µL	Normal
Total WBC	8.72 × 10⁹/L	4.00-10.00 × 10⁹/L	Normal
Neutrophils	89.1%	-	↑ Elevated
Lymphocytes	9.4%	-	↓ Reduced

Inflammatory markers (Table 2) were within normal limits at the time of assessment. The patient’s C-reactive protein (CRP) level was reported as <6 mg/L (reference: <6.0 mg/L), and the erythrocyte sedimentation rate (ESR) was 12 mm/hour (reference: 0-20 mm/hour). These values do not indicate an active systemic inflammatory response. The normal CRP and ESR support the absence of acute infectious or overt inflammatory etiology at presentation, further narrowing the differential diagnosis and aligning with a thrombotic rather than infectious process in this case of CVST.

**Table 2:** Inflammatory markers CRP - C-reactive protein; ESR - erythrocyte sedimentation rate

Test	Result	Reference Range	Interpretation
CRP	<6 mg/L	<6.0 mg/L	Normal
ESR	12 mm/hour	0-20 mm/hour	Normal

The patient's coagulation parameters (Table 3) revealed a shortened activated partial thromboplastin time (APTT) of 14.3 seconds, which is below the normal reference range of 24.6-38.6 seconds. The international normalized ratio (INR) was 1.15, remaining within the normal range (~1.0), indicating adequate baseline coagulation function. The isolated reduction in APTT may be of limited clinical significance in the absence of bleeding diathesis or concurrent anticoagulation and could reflect laboratory variability or a physiological response. These findings were considered in the context of the patient's thrombotic presentation and did not preclude the initiation of therapeutic anticoagulation with low molecular weight heparin (LMWH).

**Table 3:** Coagulation profile APTT - activated partial thromboplastin time; INR - international normalized ratio

Test	Result	Reference Range	Interpretation
APTT	14.3 seconds	24.6-38.6 seconds	↓ Low
INR	1.15	~1.0 (normal)	Normal

Serial measurements of serum sodium (Table 4) showed a mild downward trend (136.6, 134.8, and 131.0 mmol/L), with the final value falling just below the lower limit of the reference range (135-145 mmol/L), indicating mild hyponatremia. Serum potassium levels remained within normal limits across all three readings (4.7, 3.8, and 4.0 mmol/L; reference range 3.5-5.0 mmol/L), with no evidence of electrolyte imbalance requiring correction. Serum creatinine levels were consistently within the normal reference range (0.6-1.3 mg/dL), with values of 1.24, 0.90, and 0.80 mg/dL, indicating preserved renal function throughout the acute phase of illness. These findings supported the safe use of anticoagulant therapy without the need for renal dosing adjustments.

**Table 4:** Renal functions and serum electrolytes

Test	Results	Reference Range	Interpretation
Sodium	136.6 mmol/L	135-145 mmol/L	Normal
Potassium	4.7 mmol/L	3.5-5.0 mmol/L	Normal
Creatinine	0.80 mg/dL	0.6-1.3 mg/dL	Normal

Liver function parameters (Table 5) remained within normal limits throughout the course of illness. Total bilirubin was measured at 0.80 mg/dL (reference range: 0.50-1.30 mg/dL), with direct and indirect fractions at 0.17 mg/dL and 0.28 mg/dL, respectively, indicating no evidence of hepatic cholestasis or significant hemolysis. Alanine aminotransferase (ALT) and aspartate aminotransferase (AST) levels were 12.4 U/L and 13.4 U/L, respectively (reference range: 0-45 U/L), suggesting no hepatocellular injury. Alkaline phosphatase (ALP) was 89.4 U/L (reference range: 47-119 U/L), further supporting normal hepatobiliary function. These findings excluded hepatic dysfunction as a contributing factor to the clinical presentation. 

**Table 5:** Liver function tests ALP - alkaline phosphatase; ALT - alanine aminotransferase; AST - aspartate aminotransferase

Test	Result	Reference Range	Interpretation
Total bilirubin	0.80 mg/dL	0.50-1.30 mg/dL	Normal
Direct bilirubin	0.17 mg/dL	<0.30 mg/dL	Normal
Indirect bilirubin	0.28 mg/dL	Calculated	Normal
ALT	12.4 U/L	0-45 U/L	Normal
AST	13.4 U/L	0-45 U/L	Normal
ALP	89.4 U/L	47-119 U/L	Normal

Comprehensive autoimmune and thrombophilia screening (Table 6) was performed to investigate underlying etiologies for the CVST. Results showed a negative antinuclear antibody (ANA) and anti-double-stranded DNA (anti-dsDNA) antibody, effectively ruling out SLE and related connective tissue disorders. Antiphospholipid antibody testing, including anti-β2 glycoprotein I and anti-cardiolipin antibodies, was also negative, minimizing the likelihood of antiphospholipid syndrome. Serum homocysteine was within normal limits at 10 µmol/L (reference: 5-15 µmol/L), suggesting no hyperhomocysteinemia. Both cytoplasmic and perinuclear ANCA (C-ANCA and P-ANCA) were negative, excluding ANCA-associated vasculitides. Collectively, these results indicate the absence of identifiable autoimmune or prothrombotic disorders in this patient. 

**Table 6:** Autoimmune and thrombophilia panel - results and reference ranges ANCA - antineutrophil cytoplasmic antibodies; IIF - indirect immunofluorescence

Test	Result	Reference Range
Antinuclear antibody	Negative	Negative
Anti-dsDNA antibody	Negative	<30 IU/mL (negative)
Anti-β2 glycoprotein I antibody	Negative	<20 GPL/MPL units
Anti-cardiolipin antibody	Negative	<20 GPL/MPL units
Serum homocysteine	10 µmol/L	5-15 µmol/L
C-ANCA (cytoplasmic ANCA)	Negative	<1:20 (negative by IIF)
P-ANCA (perinuclear ANCA)	Negative	<1:20 (negative by IIF)

CVST is frequently associated with both acquired and inherited prothrombotic states. In patients who present with CVST without an immediately identifiable risk factor, such as infection, trauma, oral contraceptive use, or malignancy, a thorough thrombophilia workup is essential to uncover underlying etiologies and to guide long-term anticoagulation strategies. In addition, a contrast-enhanced CT of the chest, abdomen, and pelvis (CECT CAP) was arranged to exclude an underlying malignancy as a potential prothrombotic etiology.

**Figure 1:**
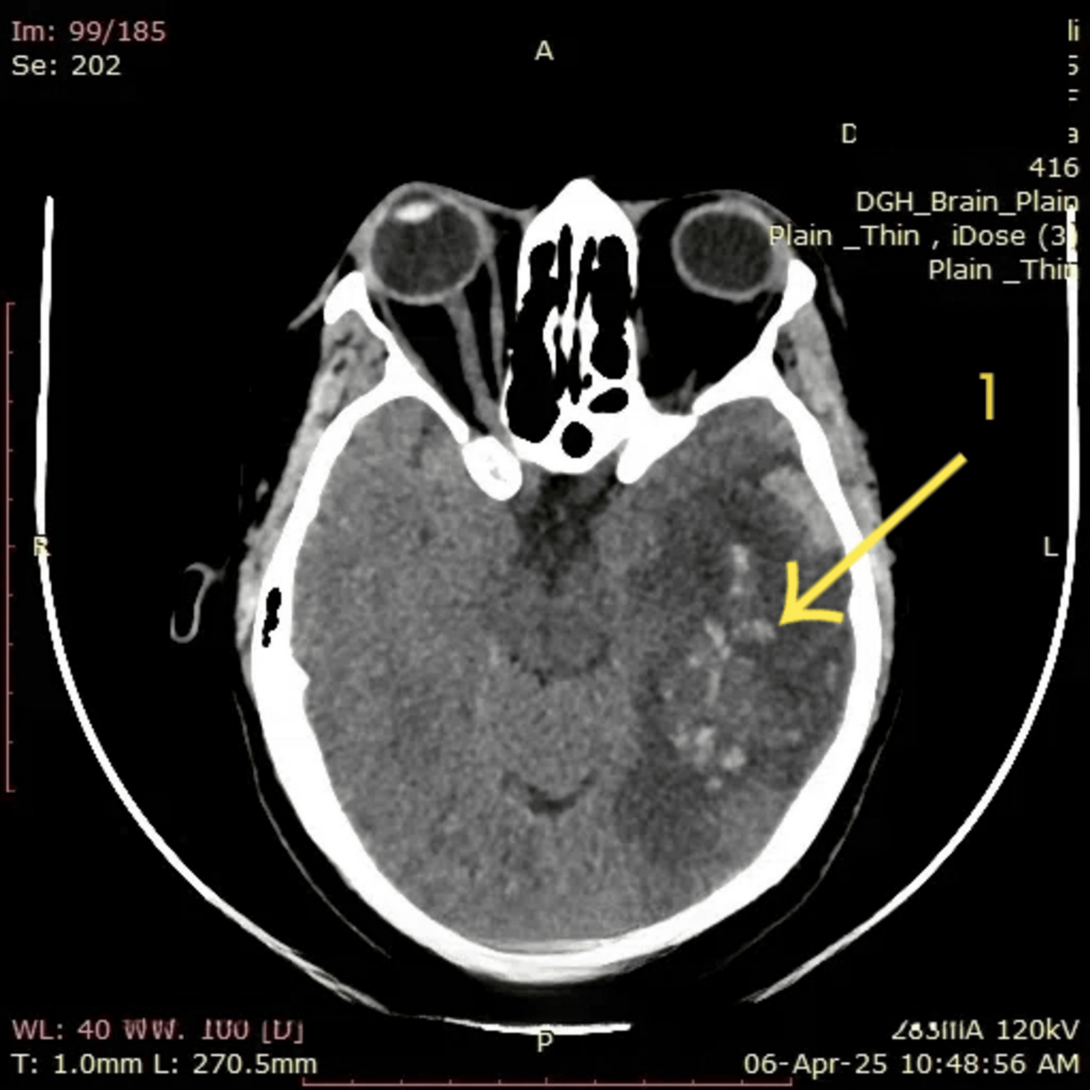
Non-contrast CT brain (05/04/2025) Arrow 1: The hemorrhagic focus is surrounded by extensive hypodense edema, consistent with venous infarction.

A NCCT of the brain (Figure 1) revealed a hyperdense lesion in the left temporoparietal region with surrounding hypodensity, suggestive of hemorrhagic venous infarction (arrow 1). The lesion demonstrated features concerning for underlying space-occupying pathology; however, no definitive mass effect or midline shift was noted. Given the atypical presentation and imaging findings mimicking tumor hemorrhage, further evaluation was warranted.

**Figure 2:**
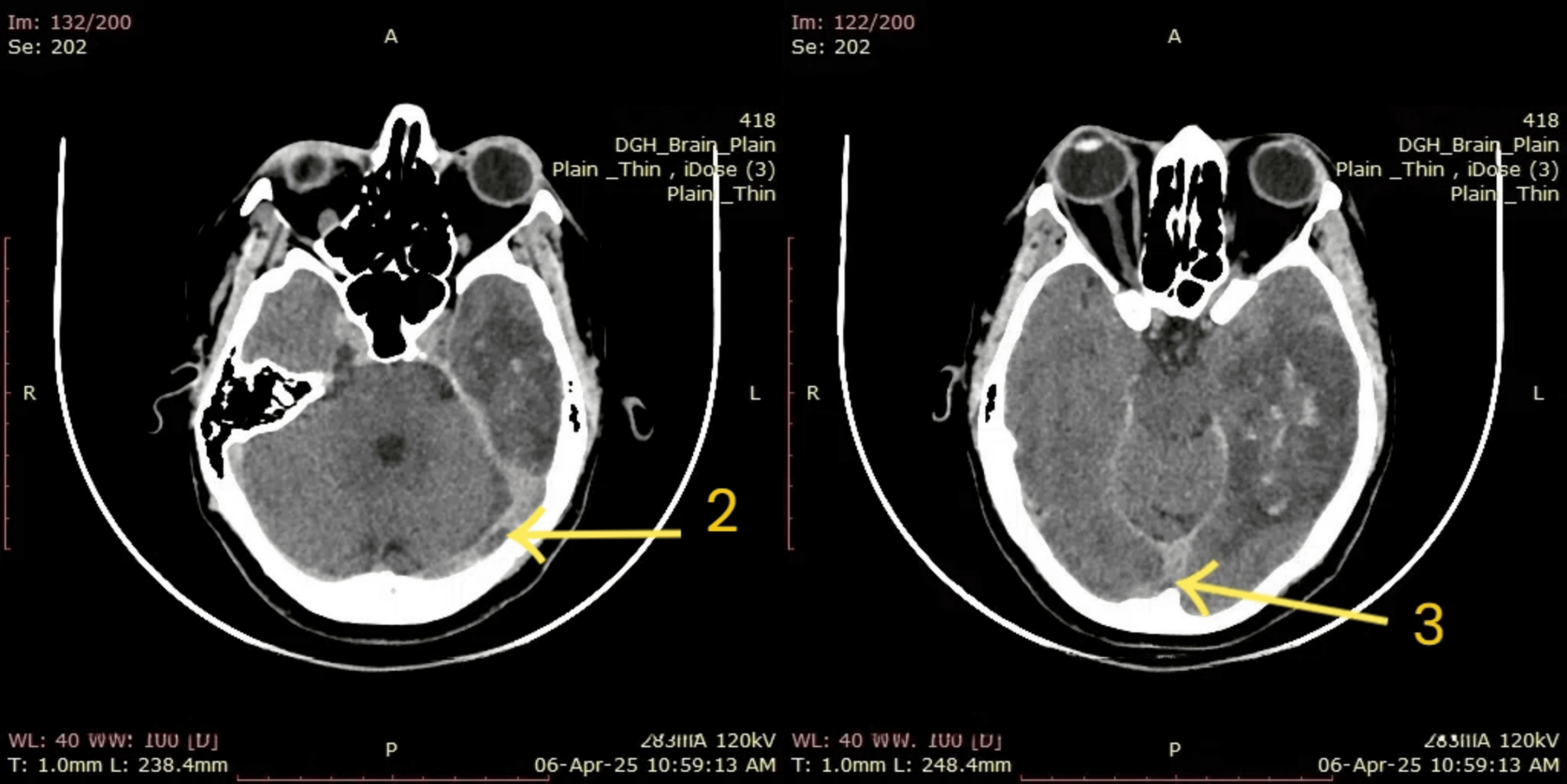
CT venogram - brain Arrow 2: There is a clear filling defect within the left transverse sinus, demonstrating a lack of opacification in comparison with the contralateral side. Arrow 3: Confluence of sinuses shows a poor contrast filling, consistent with propagation of thrombus from the transverse.

The CT venogram (Figure 2) demonstrates a filling defect within the left transverse sinus (arrow 2), consistent with thrombus formation, along with poor opacification in the confluence of sinuses (arrow 3), indicating propagation of the thrombus. Right-sided venous sinuses opacify normally. These findings are indicative of acute CVST involving the left transverse sinus.

**Figure 3:**
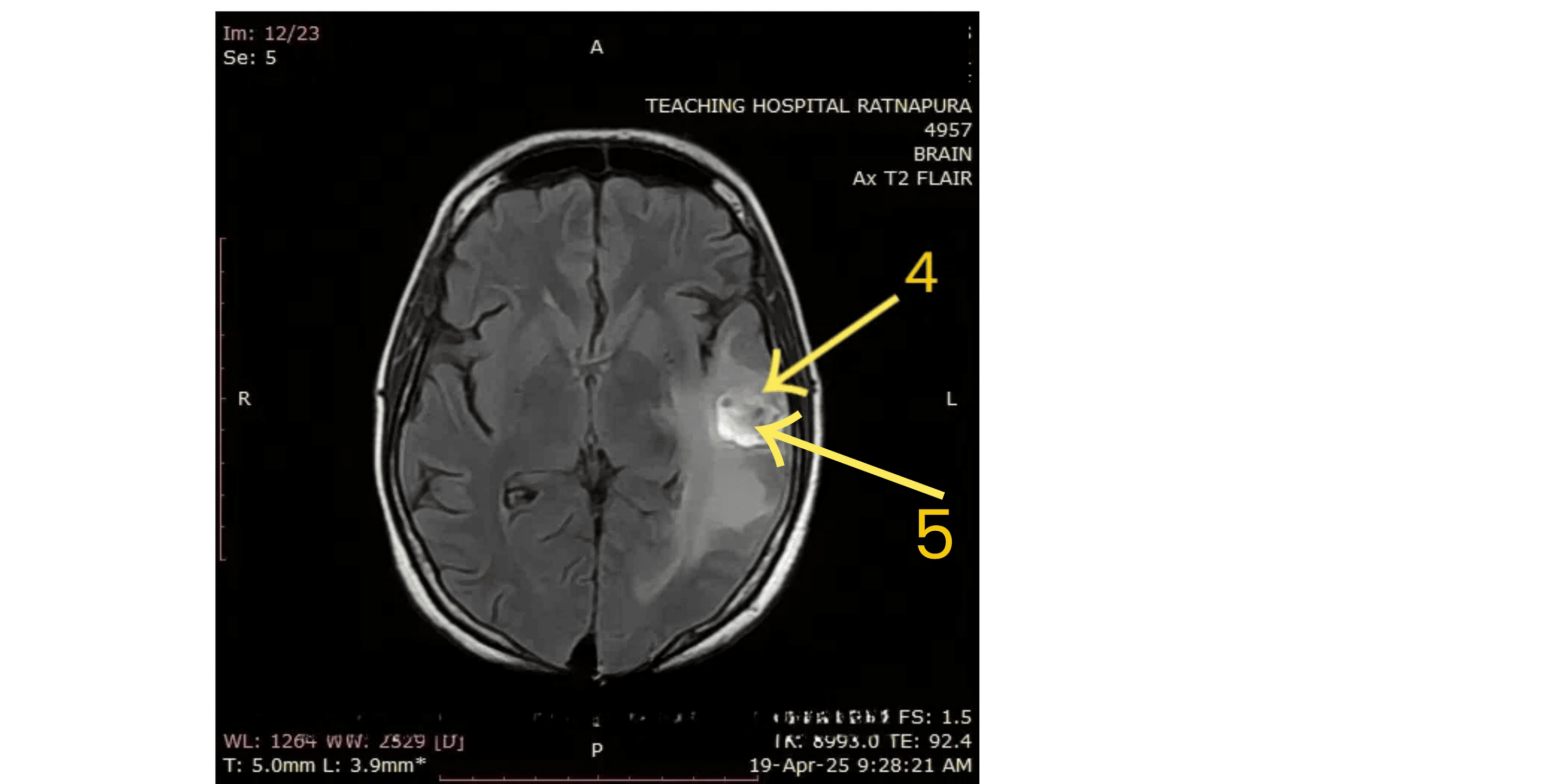
Axial Fluid-Attenuated Inversion Recovery (FLAIR) MRI Arrow 4: There is a heterogeneously hyperintense lesion in the left parietotemporal region, with a central hypointense core and surrounding hyperintense signal consistent with vasogenic edema. Arrow 5: Corresponds to the same lesion and shows it as hyperintense with a hypointense center, suggesting hemorrhagic venous infarction.

Figure 3 shows an axial Fluid-Attenuated Inversion Recovery (FLAIR) MRI sequence, demonstrating a heterogeneously hyperintense lesion in the left parietotemporal region, with surrounding vasogenic edema (arrow 4) and a hypointense center suggestive of hemorrhagic transformation (arrow 5). These findings highlight the extent of parenchymal involvement consistent with a hemorrhagic venous infarct. These findings are consistent with a hemorrhagic venous infarct secondary to CVST, likely involving the cortical veins draining the dominant hemisphere.

**Figure 4:**
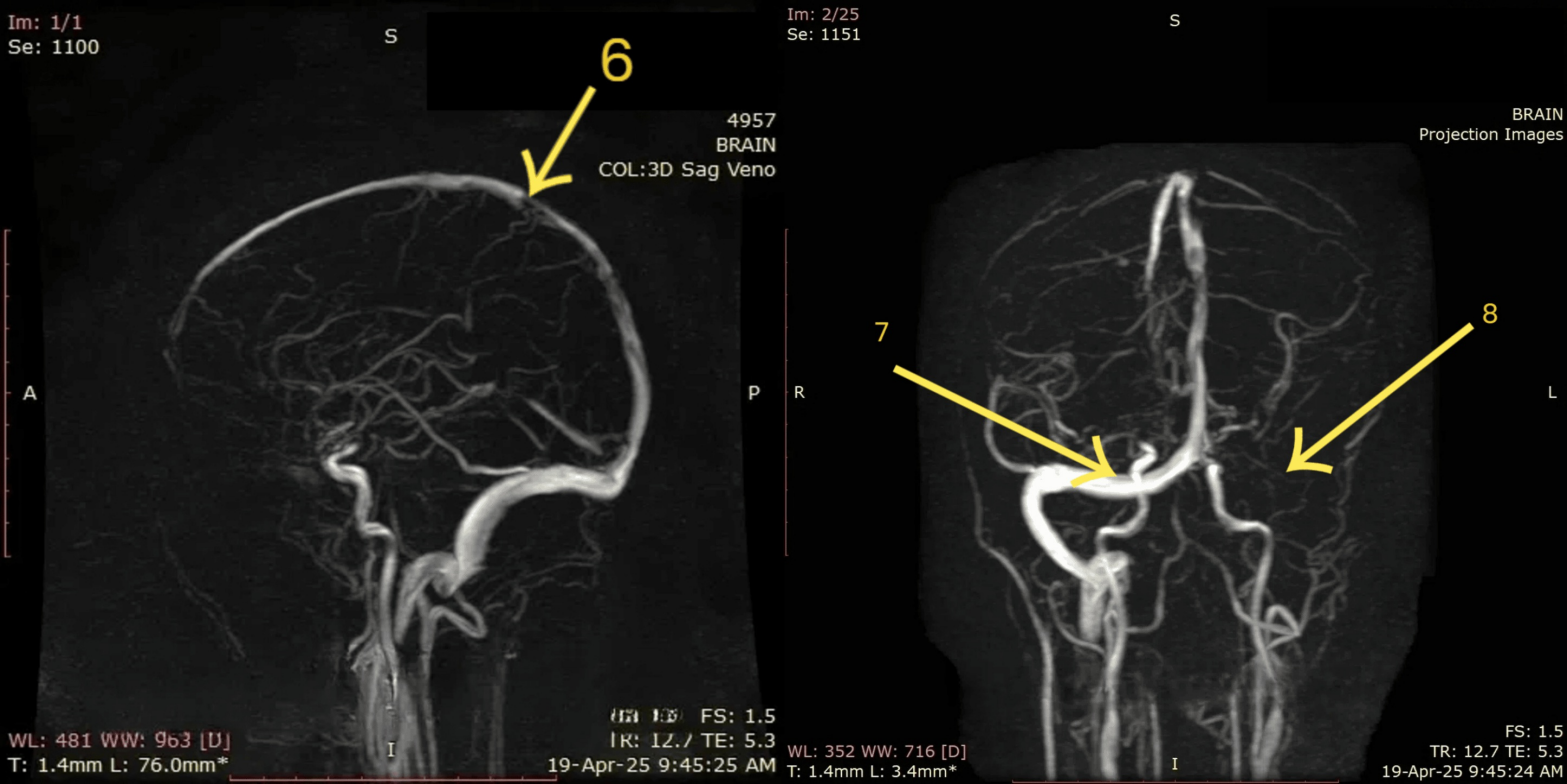
Magnetic resonance venography (MRV) Arrow 6 (left image - sagittal view): Indicates a filling defect or poor opacification in the superior sagittal sinus. Arrow 7 (right image - coronal view): Highlights normal right transverse sinus. Arrow 8 (right image): Points toward a hypoplastic or thrombosed left sigmoid sinus with reduced visualization of the corresponding cortical venous drainage.

MRV (Figure 4) demonstrates multiple filling defects along the cerebral venous sinuses. The sagittal projection (arrow 6) shows poor opacification of the superior sagittal sinus, suggestive of thrombosis. Coronal views further reveal normal right transverse sinus (arrow 7) and reduced flow-related signal intensity in the left sigmoid sinus (arrow 8), consistent with thrombotic occlusion. These imaging features, when correlated with clinical presentation and other neuroimaging findings, confirm the diagnosis of CVST predominantly affecting the left transverse and sigmoid sinuses as well as the superior sagittal sinus.

**Figure 5:**
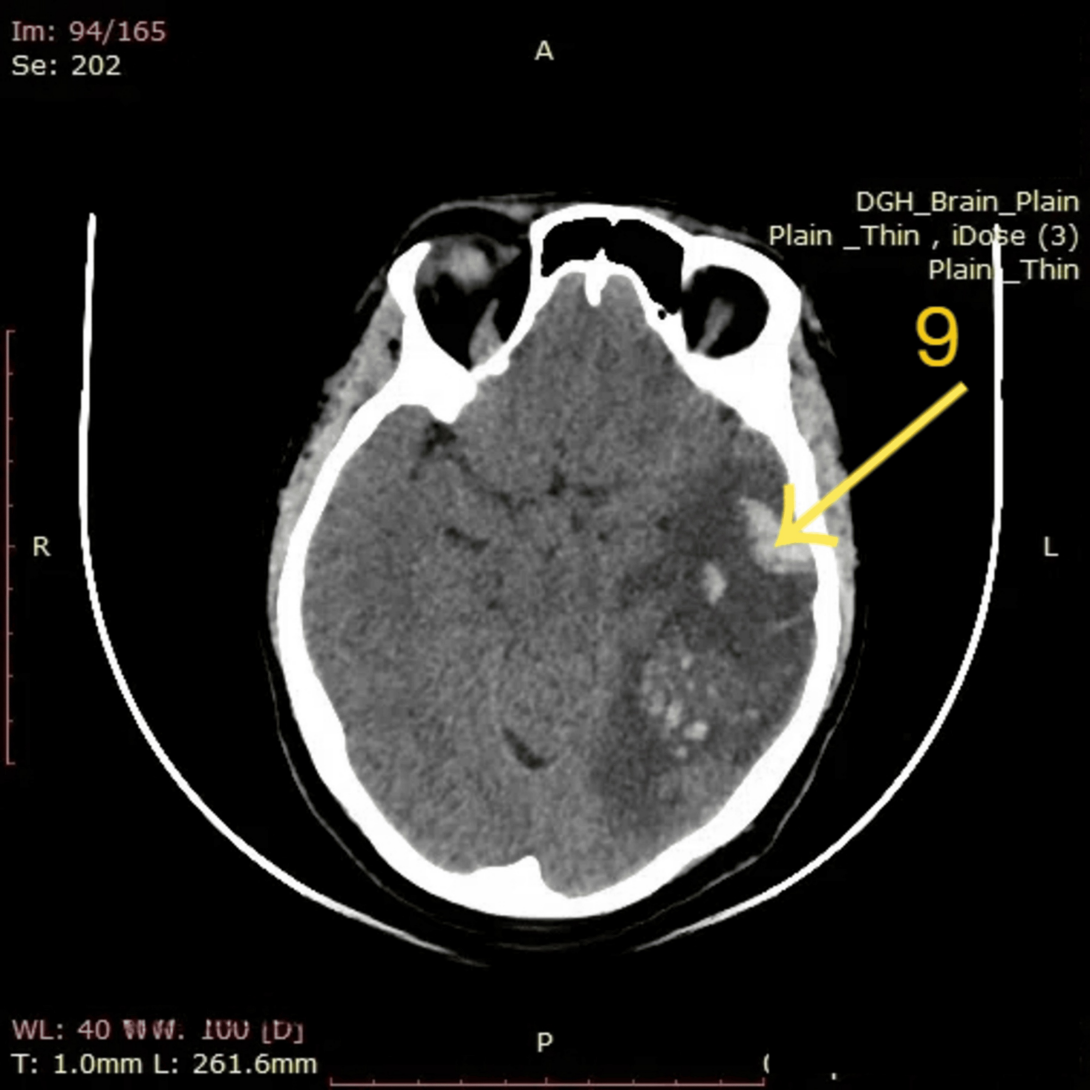
Non-contrast CT (NCCT) brain two days after starting anticoagulation Arrow 9: Well-defined hyperdense area in the left temporal lobe. This hyperdensity is consistent with resolving hemorrhagic venous infarction in the context of cerebral venous sinus thrombosis (CVST).

The initial NCCT brain image (Figure 1) shows an area of hyperdensity in the left temporal lobe (arrow 1) suggestive of hemorrhagic venous infarction, with surrounding vasogenic edema and mass effect evidenced by compression of adjacent sulci. On follow-up NCCT two days after starting anticoagulation (Figure 5), the hyperdense focus remains visible (arrow 9); however, there is no radiological evidence of hemorrhagic progression. The mass effect appears slightly reduced, and no new hemorrhagic transformation is noted. These imaging findings, when correlated with the patient’s clinical improvement, suggest radiological stability and favorable response to anticoagulation therapy initiated for CVST.

The patient was initially managed with LMWH (enoxaparin), followed by bridging therapy with warfarin, which was commenced on day five of admission. The patient remained clinically stable and continued to improve throughout hospitalization. She was discharged after two weeks, once therapeutic anticoagulation was achieved with a target INR range of 2-3. At discharge, she was neurologically intact, with complete resolution of aphasia and no residual deficits. Outpatient follow-up was arranged for two weeks post-discharge to reassess clinical status and repeat INR monitoring.

## Discussion

CVST is a rare but potentially reversible cause of stroke, accounting for approximately 0.5-1% of all strokes. Unlike arterial strokes, which typically present with sudden-onset focal neurological deficits, CVST often manifests with nonspecific symptoms, resulting in diagnostic delays. Headache is the most common presenting symptom, followed by focal deficits, seizures, and signs of raised intracranial pressure such as papilledema [1,2].

In the present case, the patient exhibited progressive expressive aphasia and headache, without classical stroke features such as hemiparesis or facial asymmetry. While aphasia is more commonly associated with arterial infarcts, it can be an underrecognized presentation in CVST, particularly when thrombosis involves veins draining the dominant hemisphere, typically the left frontal, temporal, or parietal lobes [1]. Common venous sites implicated in language dysfunction include the left transverse sinus, left sigmoid sinus, superior sagittal sinus, and cortical veins, such as the vein of Labbé, which drains the lateral temporal lobe [16].

The type of aphasia observed depends on the site of involvement. Thrombosis affecting the left inferior frontal gyrus may cause Broca’s aphasia, while Wernicke’s aphasia may result from involvement of the left superior temporal gyrus. More extensive infarction may produce global aphasia, and transient aphasia may occur due to edema or increased intracranial pressure, even in the absence of infarction [2,5].

Several case reports have documented expressive aphasia as a presenting feature of CVST. One example describes a 27-year-old male with small cell lung cancer who developed Broca’s aphasia secondary to left transverse sinus thrombosis following chemotherapy. Another case involved a 38-year-old postpartum woman with mixed transcortical aphasia due to extensive dural sinus thrombosis. These reports highlight the need to consider CVST in patients presenting with unexplained aphasia, especially when associated with prothrombotic risk factors such as malignancy or postpartum status.

In this patient, NCCT initially revealed hemorrhagic transformation of a venous infarct, radiologically mimicking tumor hemorrhage or bleeding into an old infarct (Figure 1). This diagnostic dilemma has been echoed in other reports. For instance, a 20-year-old woman presented with headache and left-sided weakness, and NCCT showed hypodense lesions with hyperdense regions in the right frontoparietal lobes, raising suspicion for glioma hemorrhage. However, MRV (Figure 2 and Figure 3) later confirmed cortical venous infarction due to CVST [19]. Another young woman with cervical cancer presented with seizures and was initially suspected to have a tumor bleed; imaging later confirmed hemorrhagic CVST [20]. Similarly, a case of isolated cerebellar vein thrombosis mimicked a space-occupying lesion [21].

In our case, the initial radiological interpretation also favored tumor hemorrhage, underscoring the diagnostic complexity of CVST. However, a subsequent CT venogram revealed thrombosis of the dural venous sinuses, establishing the correct diagnosis. This highlights the importance of venous phase imaging in patients with atypical CT findings or unexplained neurological symptoms.

Despite the presence of hemorrhagic venous infarction, anticoagulation remains the cornerstone of CVST management. Randomized trials and observational studies have shown that anticoagulation with LMWH prevents thrombus propagation and promotes recanalization, with a favorable risk-benefit profile, even in the presence of hemorrhage [22,23]. The safety of LMWH in hemorrhagic CVST has been supported by other cases as well. For example, three patients - a 16-year-old girl with severe headache and seizures, a 35-year-old woman with COVID-19-related neurological deficits, and another adolescent with crescent-shaped intracranial hemorrhage - were successfully treated with enoxaparin, without worsening of the hemorrhage [24,25].

Our patient with CVT and associated hemorrhagic venous infarction was successfully managed with LMWH, specifically enoxaparin. Despite the presence of intracerebral hemorrhage, the patient tolerated anticoagulation well, with no evidence of clinical deterioration or radiological progression of the hemorrhage on follow-up imaging. This outcome aligns with current evidence suggesting that anticoagulation remains the cornerstone of CVT management, even in the setting of hemorrhagic transformation.

In our case, the early initiation of LMWH contributed to the stabilization of neurological symptoms and prevented further thrombus propagation. This reinforces the recommendation for prompt anticoagulation in CVT, tailored to the individual patient's clinical status and bleeding risk.

This case also emphasizes that CVST can occur in individuals without identifiable prothrombotic risk factors, reinforcing the importance of performing a thorough thrombophilia screen. Clinicians should maintain a high index of suspicion for CVST in patients with unusual neurological presentations, particularly when neuroimaging is atypical or suggests alternative pathologies. Timely diagnosis and appropriate treatment can significantly improve outcomes, which are generally favorable when intervention is initiated early.

## Conclusions

This case highlights the diagnostic challenge posed by CVST, particularly when it presents with atypical radiological findings that mimic other intracranial pathologies such as tumor hemorrhage or hemorrhagic transformation of an infarct. Despite radiological evidence of hemorrhagic venous infarction, our patient was safely and successfully treated with LMWH, in line with current evidence-based guidelines.

A high index of suspicion, timely neuroimaging with venous phase studies, and the decision to initiate anticoagulation despite hemorrhage were critical to the favorable outcome in this case. This report reinforces the importance of considering CVST in the differential diagnosis of stroke in young to middle-aged patients, especially those presenting with nonspecific symptoms and atypical imaging. Ultimately, early recognition and appropriate anticoagulation remain the cornerstone of successful CVST management, even in the presence of intracranial hemorrhage.
